# The Optimization of Pair Distribution Functions for the Evaluation of the Degree of Disorder and Physical Stability in Amorphous Solids

**DOI:** 10.3390/molecules29102379

**Published:** 2024-05-18

**Authors:** Zhihui Yuan, Zunhua Li, Jie Luo, Asad Nawaz, Bowen Zhang, Wubliker Dessie

**Affiliations:** 1 College of Chemistry and Bioengineering, Hunan University of Science and Engineering, Yongzhou 425199, China; zhh_yuan@126.com (Z.Y.); luojie@huse.edu.cn (J.L.); 007298@yzu.edu.cn (A.N.); dwubliker@huse.edu.cn (W.D.); 2 School of Chemical Engineering and Technology, Tianjin University, Tianjin 300072, China; bowen_667@tju.edu.cn

**Keywords:** amorphous drug, physical stability assessment conditions, pair distribution function, principal component analysis

## Abstract

The amorphous form of poorly soluble drugs is physically unstable and prone to crystallization, resulting in decreased solubility and bioavailability. However, the conventional accelerated stability test for amorphous drugs is time-consuming and inaccurate. Therefore, there is an urgent need to develop rapid and accurate stability assessment technology. This study used the antitumor drug nilotinib free base as a model drug. The degree of disorder and physical stability in the amorphous form was assessed by applying the pair distribution function (PDF) and principal component analysis (PCA) methods based on powder X-ray diffraction (PXRD) data. Specifically, the assessment conditions, such as the PDF interatomic distance range, PXRD detector type, and PXRD diffraction angle range were also optimized. The results showed that more reliable PCA data could be obtained when the PDF interatomic distance range was 0–15 Å. When the PXRD detector was a semiconductor-type detector, the PDF data obtained were more accurate than other detectors. When the PXRD diffraction angle range was 5–40°, the intermolecular arrangement of the amorphous drugs could be accurately predicted. Finally, the accelerated stability test also showed that under the above-optimized conditions, this method could accurately and rapidly assess the degree of disorder and physical stability in the amorphous form of drugs, which has obvious advantages compared with the accelerated stability test.

## 1. Introduction

Various solid-state forms of pharmaceutical active ingredients (APIs) exhibited distinct physical and chemical properties, particularly concerning solubility and bioavailability, due to their specific internal molecular stacking structures. Based on their low solubility, the biopharmaceutical classification system (BCS) categorized these drugs into classes II and IV. It has been estimated that approximately 40% of drugs currently available on the market and 70–90% of APIs in the developmental pipeline belong to these classes [1]. Consequently, there has been a notable emphasis on enhancing the solubility of drugs and improving their bioavailability through the utilization of the amorphous state. For example, the apparent solubility of lumefantrine and the α-chitin amorphous form can be significantly enhanced, increasing the bioavailability of these drugs [2,3].

Amorphous drugs are known to exhibit instability and a propensity to transform into a crystalline form, resulting in reduced solubility and bioavailability [4]. This phenomenon was especially evident when amorphous drugs were subjected to high temperature and humidity [5,6]. Therefore, the development of a technology for assessing the physical stability in amorphous drugs is of paramount importance. The current approach to evaluating the physical stability in amorphous drugs involves conducting either an accelerated stability test, which entails subjecting the drugs to 40 °C and 75% relative humidity for 6 months, or a long-term stability test, which involves exposing the drugs to 25 °C and 60% relative humidity for 2 years [7]. These tests were conducted to determine if there have been any alterations in the physical properties of the amorphous drugs. The assessment process, however, is characterized by its time-consuming and labor-intensive nature. Moreover, the X-ray powder diffraction (PXRD) patterns of amorphous drugs displayed a solitary halo peak, posing challenges in distinguishing different amorphous samples solely based on their PXRD patterns [8,9]. Therefore, PXRD is not deemed sufficiently accurate for evaluating the physical stability in amorphous drugs.

Hence, the development of a novel technique for assessing the physical stability in amorphous drugs is of utmost importance. Interestingly, the pair distribution function (PDF) obtained through Fourier transformation of the PXRD data served as a valuable tool for the structural analysis of amorphous drugs [10]. This method offered valuable insights into the structural characteristics of amorphous materials by providing information on short-range order, medium-range order, and long-range order [11]. For instance, a quantitative depiction of the local structure at different grinding stages was obtained through the analysis of the PDF data of ground samples containing α, γ, and δ crystalline forms of indomethacin [12]. As indicated by the PDF, at a specific distance (r), the peak intensity (G(r)) of the ordered crystalline form is significantly greater than that of the disordered amorphous form [13]. Additionally, Davis et al. presented a methodology for the identification and quantification of phase fractions in cryo-milled sulfamerazine through the utilization of PDF analysis [14]. Therefore, the degree of disorder exhibited by various forms can be evaluated through the analysis of the respective PDF data.

Moreover, the PDF analysis also offered valuable insights into the evaluation of the physical stability, specifically the time it takes for amorphous materials to undergo crystallization. For example, the PDF analysis can evaluate the optimal cryo-milling duration required to achieve the maximum degree of disorder and physical stability in the indomethacin samples [15]. Naelapää et al. conducted a PDF analysis to elucidate the recrystallization behavior, specifically the physical stability, of amorphous piroxicam samples [16]. These two studies demonstrated a positive correlation between the degree of order in amorphous samples and the likelihood of recrystallization. This finding suggested that the physical stability in the amorphous samples decreased as the degree of order increased. By analyzing the PDF, valuable insights can be obtained regarding the degree of disorder and the physical stability in amorphous samples.

Nowadays, principal component analysis (PCA) has become a valuable tool for obtaining qualitative and quantitative information on amorphous drugs. It was used, for instance, in amorphous preparation [17] and crystalline-to-amorphous phase transformations [18]. With the aid of PCA analysis, subtle differences that are not visible to the human eye can easily be distinguished [19]. So, the preparation parameters for obtaining disorder and physically stable amorphous drugs can be optimized based on PCA results. This method for assessing the degree of disorder and physical stability can be conducted immediately after preparing amorphous samples, without requiring excessive time or labor. Therefore, it is an accurate, fast, and convenient method for assessing the degree of disorder and physical stability in amorphous drugs.

Nilotinib free base is an antitumor drug used in clinical settings to treat chronic myeloid leukemia that is resistant to imatinib [20]. However, one of the most significant drawbacks of the crystalline form of nilotinib free base is its low solubility, which results in low bioavailability [21]. To address this issue, various formulations have been developed to significantly enhance the solubility of nilotinib free base. These includes a spray-dried solid dispersion, an amorphous form, and a nanoparticle formulation containing an amorphous form of the drugs [22,23,24]. In addition, a supersaturating drug delivery system, a self-micro-emulsifying drug delivery system, and surfactant-based formulations have been developed to enhance the oral bioavailability of nilotinib free base [25,26,27].

Accordingly, a nilotinib free base was used as the model drug in this study. Firstly, the crystal structure of nilotinib free base crystalline Form A was described. Secondly, a novel technology was developed to assess the degree of disorder and physical stability in amorphous drugs by applying the PDF and PCA methods based on PXRD data. Thirdly, the assessment conditions were optimized. The optimized assessment conditions include the PDF interatomic distance range, the PXRD detector type, and the PXRD diffraction angle range. Finally, the optimized assessment conditions were verified by the accelerated stability test experiments.

## 2. Results and Discussion

### 2.1. Optimization of PDF Interatomic Distance Range

#### 2.1.1. Interatomic Distance of Nilotinib Free Base Molecule

**Figure 1:**
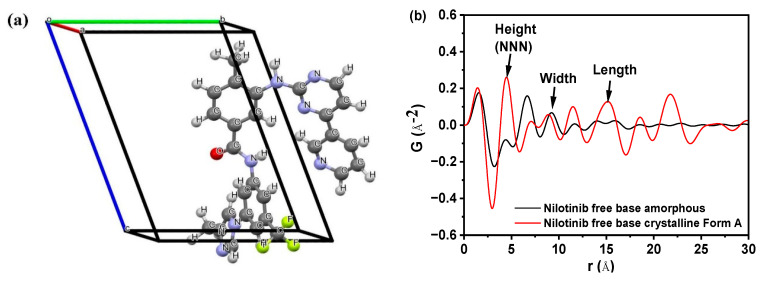
(**a**) Crystal structure of nilotinib free base crystalline Form A (Reproduced with permission from [28]. Copyright 2015 JCPDS-ICDD); (**b**) comparing the PDF of nilotinib free base crystalline Form A and amorphous form.

Figure 1a demonstrated the crystal structure of nilotinib free base crystalline Form A [28]. This form crystallized in space group *P*1, with lattice parameters *a* = 4.52 Å, *b* = 10.64 Å, *c* = 13.70 Å, *α* = 68.86°, *β* = 82.15°, *γ* = 84.20°, volume *V* = 607.62 Å^3^, and *Z* = 1. The dimensions of the crystal lattice in the nilotinib free base crystalline Form A were determined to be 4.52, 10.64, and 13.70 Å for the height, width, and length, respectively. Figure 1b displayed several peaks in the PDF that correspond to the interactions between nearest neighbors. A comparison was made between the crystalline Form A of nilotinib free base and its amorphous form. The analysis revealed significant alterations in the local nearest neighbor molecular configuration, suggesting a transition from a crystalline to an amorphous packing structure. The arrows indicated that the three nearest neighbor peaks correspond to atom-to-atom distances of 4.62 Å, 9.43 Å, and 14.35 Å. Additionally, the nilotinib free base crystal lattice in the crystalline Form A structure exhibited dimensions of 4.52 Å in height, 10.64 Å in width, and 13.70 Å in length. These measurements closely align with the three nearest neighbor distances determined from the PDF data. The distances observed suggest that the average local structure of the amorphous phase of nilotinib free base was randomly close-packed and influenced by the anisotropy of the molecular shape.

Additionally, in the comparison of the PDF data of the amorphous form and crystalline Form A of the nilotinib free base, it was observed that crystalline Form A exhibited greater fluctuations than the amorphous form. The intensity of the second peak (also known as the next nearest neighbor (NNN) peak, G_NNN_, which corresponds to the crystal lattice height of the nilotinib free base crystalline Form A) in the amorphous form was significantly lower compared to the intensity of the second peak in crystalline Form A. According to the definition of PDF, the G_NNN_ value represents the degree of disorder between an atom and its next nearest neighbor in the solid samples. A low G_NNN_ value indicates a high degree of disorder in the solid samples, whereas a high G_NNN_ value indicates a low degree of disorder. Therefore, we can ascertain the disorder with high certainty simply by comparing the G_NNN_ values of various samples. This observation suggested that the degree of disorder in the amorphous form was significantly lower compared to that in the crystalline Form A. Similar findings have been reported in prior research conducted on other drugs. For example, it has been deduced that the first and second peaks observed in the PDF can be attributed to the γ crystalline form of indomethacin, representing the molecular height and width, respectively. Additionally, the third peak in the PDF corresponds to the γ crystalline form of indomethacin, specifically indicating the molecular length of the dimer [13,15]. In the PDF of piroxicam, the distances of 12.3 Å, 7.9 Å, and 4.9 Å correspond to the crystal lattice dimensions (height, width, and length, respectively) of piroxicam in its crystalline form [29].

#### 2.1.2. Using PCA to Compare between Various Amorphous Solids

**Figure 2:**
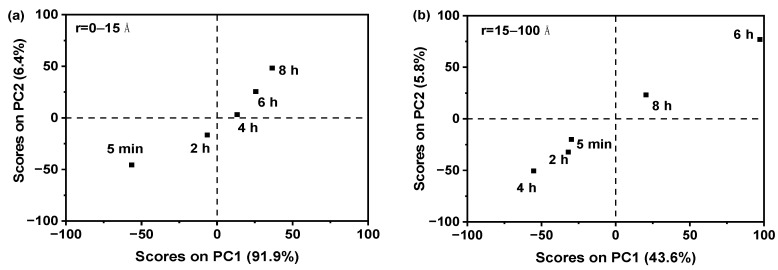
The effect of PDF interatomic distance range on PCA of nilotinib free base amorphous: (**a**) PCA (r = 0–15 Å); (**b**) PCA (r = 15–100 Å).

During the PCA analysis of the PDF data, it is crucial to utilize the appropriate PDF interatomic distance range. If an inaccurate PDF interatomic distance range is utilized in the construction of the PCA, the outcomes may yield erroneous information regarding the characteristics of amorphous drugs. Therefore, to ensure the accuracy and reliability of the PCA data obtained through PDF analysis, this study employed varied PDF interatomic distance ranges for PCA analysis. As shown in Figure 2a, utilizing a PDF interatomic distance range of 0–15 Å, it was observed that the PCA scores on PC1 exhibited a gradual increase with the aging time. This change pattern was evident and consistent. However, when employing a PDF interatomic distance range of 15–100 Å (Figure 2b), the PCA scores on PC1 did not exhibit a discernible pattern of change with increasing aging time. The obtained results indicated that the PCAs based on the PDF interatomic distance range of 0–15 Å were found to be more reliable compared to the range of 15–100 Å.

The PXRD signal for the amorphous solid is predominantly concentrated within the distance range of 0–15 Å. This observation has been corroborated by previous studies [30]. This phenomenon occurred as a result of the inclusion of short-range order and medium-range order information within the range of 0–15 Å in the amorphous materials [31]. The selected range of 15–100 Å successfully captured the intricate details of long-range order in the samples. However, in the case of amorphous materials, the absence of long-range order poses a challenge to accurately represent the disordered arrangement observed in the amorphous form samples using PCA results. Therefore, it was considered more dependable to select PDF data containing short-range order and medium-range order information within the range of 0–15 Å for the PCA analysis of amorphous samples.

#### 2.1.3. Using PCA to Compare Amorphous Solids and Crystalline Form A

**Figure 3:**
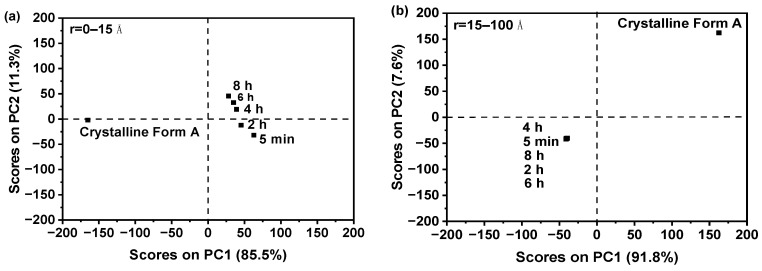
The effect of PDF interatomic distance range on the PCA of nilotinib free base amorphous and crystalline: (**a**) PCA (r = 0–15 Å); (**b**) PCA (r = 15–100 Å).

Figure 3a illustrated that the PCAs of amorphous samples of nilotinib free base, prepared at various aging times, exhibited significant differences from the PCA of crystalline Form A (used as a reference) when the PDF interatomic distance range was between 0 and 15 Å. With the increase in aging time, the scores on PC1 exhibited a progressive convergence towards the crystalline Form A. This suggested that the degree of order in the amorphous sample, prepared under a prolonged aging time, became increasingly comparable to that of the crystalline Form A. However, when the PDF interatomic distance range was set to 15–100 Å (Figure 3b), the PCAs of the amorphous samples of nilotinib free base, prepared at various aging times, showed significant differences compared to the PCA of crystalline Form A. The PCA values of all amorphous samples were found to be mixed and could not be distinguished. It was impossible to identify differences between these amorphous samples.

Furthermore, it can be observed from this phenomenon that the PCA differences between different samples were more pronounced when the range of 0–15 Å was utilized, as opposed to when the range of 15–100 Å was employed. The reason for this could be analogous to the elucidation provided in Section 2.1.2. Therefore, in the comparison between amorphous and crystalline Form A, it was deemed more reliable to select a PDF interatomic distance range of 0–15 Å for the PCA analysis of the amorphous samples.

#### 2.1.4. Thermogravimetric Analysis (TGA)

**Figure 4:**
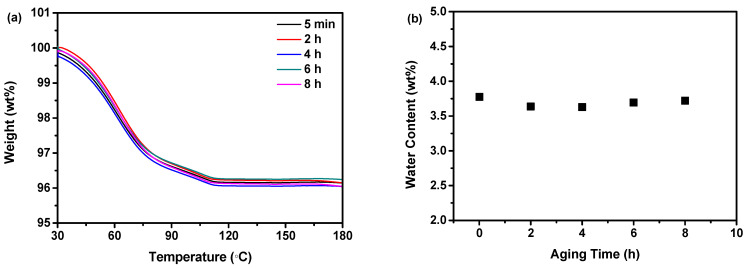
(**a**) Thermogravimetric analysis; (**b**) water content.

Figure 4a in the study under discussion presents critical insights into the thermal stability and composition of the analyzed samples. The thermogravimetric analysis (TGA) depicted a consistent pattern where the sample weights diminished as the temperature was elevated up to 100 °C. This observation can be directly linked to the evaporation of water molecules that were retained within the structure of the dried samples even after the drying process. It is important to note that water content in samples can influence various properties and behaviors, making this finding significant for further analysis and interpretation of the sample characteristics.

The TGA curve beyond 100 °C revealed a stabilization in the weight loss, indicating that the temperature had surpassed the boiling point of water, which is a crucial detail in understanding the thermal characteristics of the samples.

Furthermore, the data indicated that irrespective of the aging time—whether the samples were aged for 5 min or extended to 8 h—the weight loss was nearly identical across all samples. This uniformity strongly suggests that the water content remained constant regardless of the aging time. Such a finding is particularly relevant when considering the potential impact of aging time on the sample’s physical or chemical properties; in this context, it appears that aging time does not significantly influence water content.

This hypothesis is further corroborated by the quantitative analysis presented in Figure 4b, which calculates the water content percentage for each set of samples. According to this data, the water content remained approximately at 3.5% for all samples, despite the varied aging times. The minor fluctuations observed were within the range of experimental error and did not present a statistically significant difference, reinforcing the notion that the length of aging time did not substantially affect the amount of water retained within the samples.

In conclusion, the observations made from Figure 4a and the supporting quantitative data from Figure 4b provide valuable insights into the thermal stability and water retention properties of the studied samples. These findings suggest that the samples undergo a consistent and predictable thermal decomposition process related to water evaporation, with no significant influence from varying aging times. The consistent water content across different aging periods also implies that the sample preparation method employed is robust and delivers reproducible results, which is essential for drawing reliable conclusions from further analyses.

### 2.2. Optimization of PXRD Detector Type

**Figure 5:**
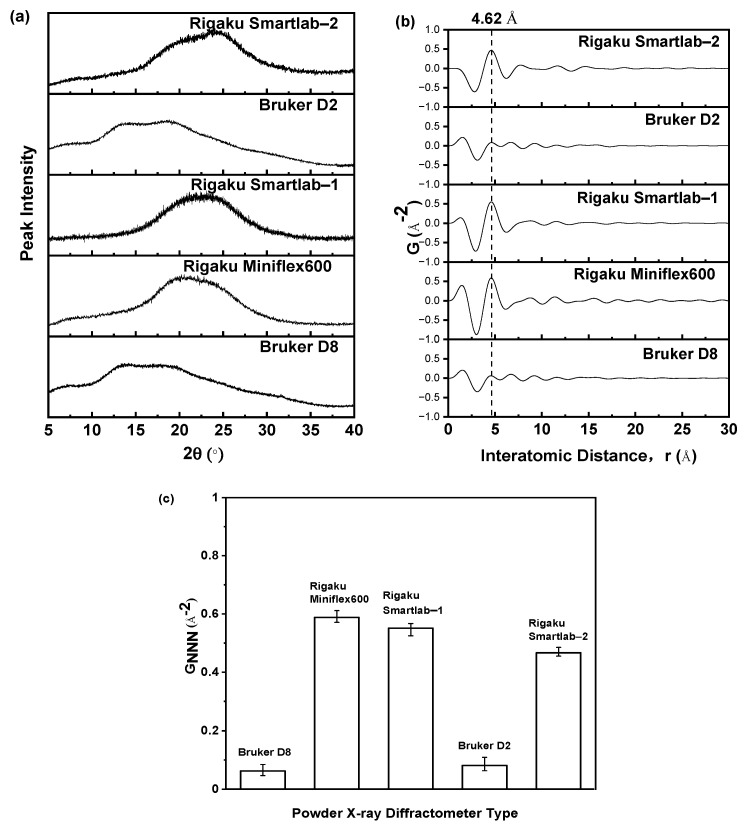
Effect of different PXRD detectors on PDF analysis: (**a**) PXRD patterns of nilotinib free base amorphous sample; (**b**) PDFs of nilotinib free base amorphous sample; (**c**) G_NNN_ (r = 4.62 Å, *n* = 3) of nilotinib free base amorphous sample. The nilotinib free base amorphous sample in these figures was prepared with an aging time of 5 min, and the PXRD diffraction angle range was 5–40°.

When conducting PXRD measurements, it is imperative to employ a suitable PXRD detector to acquire accurate PXRD patterns. If an inaccurate PXRD detector is used to obtain PXRD patterns, the results may provide misleading information about the properties of amorphous drugs. Therefore, to ensure the accuracy and reliability of the PXRD patterns obtained from PXRD measurement, this study employed a variety of PXRD detector types to determine the most suitable type. Figure 5 demonstrated the PXRD patterns acquired from various PXRD detectors for the identical amorphous sample of nilotinib free base, which was prepared with a 5-min aging time. Figure 5a demonstrated that the PXRD patterns obtained using different PXRD detectors exhibited variations due to the differing energy resolutions of the detectors. The Bruker D8 and D2 powder X-ray diffractometers were both equipped with semiconductor detectors, resulting in similar PXRD patterns. In contrast, the Rigaku Miniflex600 and Smartlab X-ray diffractometers were both equipped with scintillation detectors, resulting in similar PXRD patterns. The PXRD patterns obtained using semiconductor detectors exhibited significant differences compared to the PXRD patterns obtained using scintillation detectors.

However, a mere comparison of the different PXRD patterns was insufficient to determine the superiority of any particular detector. Therefore, it was imperative to conduct a comparative analysis of the PDF data derived from PXRD data transformation to discern the strengths and weaknesses of the detectors. The Fourier transformation was applied to the PXRD data to examine the impact of various PXRD detectors on the analysis of the PDF. As presented in Figure 5b, the peak in the PDF characteristic corresponding to the crystal lattice height of the nilotinib free base crystalline Form A was observed at 4.62 Å. It was observed that the characteristic peak intensity in the PXRD patterns obtained with the semiconductor detectors (Bruker D8 and D2 powder X-ray diffractometers) was lower compared to that obtained with the scintillation detectors (Rigaku Miniflex600 and Smartlab powder X-ray diffractometers). This suggested that the scintillation detectors exhibited a higher level of noise containment in the PXRD patterns compared to the semiconductor detectors.

As shown in Figure 5c, the G_NNN_ values (representing the G value of the next nearest neighbor, which corresponds to the crystal lattice height of the nilotinib free base crystalline Form A) of the amorphous samples, as measured by scintillation detectors, consistently exhibited higher values compared to those measured by semiconductor detectors. These G_NNN_ values correspond to the G value of the nilotinib free base crystalline Form A’s crystal lattice height or indicate the G value at an interatomic distance of 4.62 Å.

This phenomenon was observed due to the limited capability of the scintillation detector in distinguishing artefact information, such as fluorescence, present in the PXRD measurements. In contrast, the semiconductor detector demonstrated effective discrimination of these artefact information. Therefore, previous research [32,33,34] has confirmed that the utilization of a semiconductor detector for PXRD measurements, followed by Fourier transformation into PDF analysis, yields more accurate and reliable data. The energy resolution refers to the capability of a PXRD detector to differentiate between radiation with similar, yet distinct, energies. The detector’s ability to differentiate between two closely spaced radiations with similar energies improves as the energy resolution value decreases [32]. Different detectors exhibited varying levels of energy resolution. For instance, scintillation, semiconductor, and intrinsic Ge detectors exhibited energy resolutions of 40%, 2%, and 2% at 10 keV, respectively [33]. The performance of scintillation detectors was found to be subpar, while semiconductor and intrinsic Ge detectors exhibited excellent performance [34]. Therefore, when compared to the semiconductor detector and the intrinsic Ge detector, the PXRD patterns obtained using the scintillation detector were not suitable for precise PDF analysis. This was due to the poor energy resolution of the scintillation detector, which hinders its ability to effectively differentiate the sample signal from the noise signal. Hence, it is recommended to utilize semiconductor detectors as the preferred detectors for obtaining precise PXRD patterns to ensure accurate PDF analysis.

### 2.3. Optimization of PXRD Diffraction Angle Range

**Figure 6:**
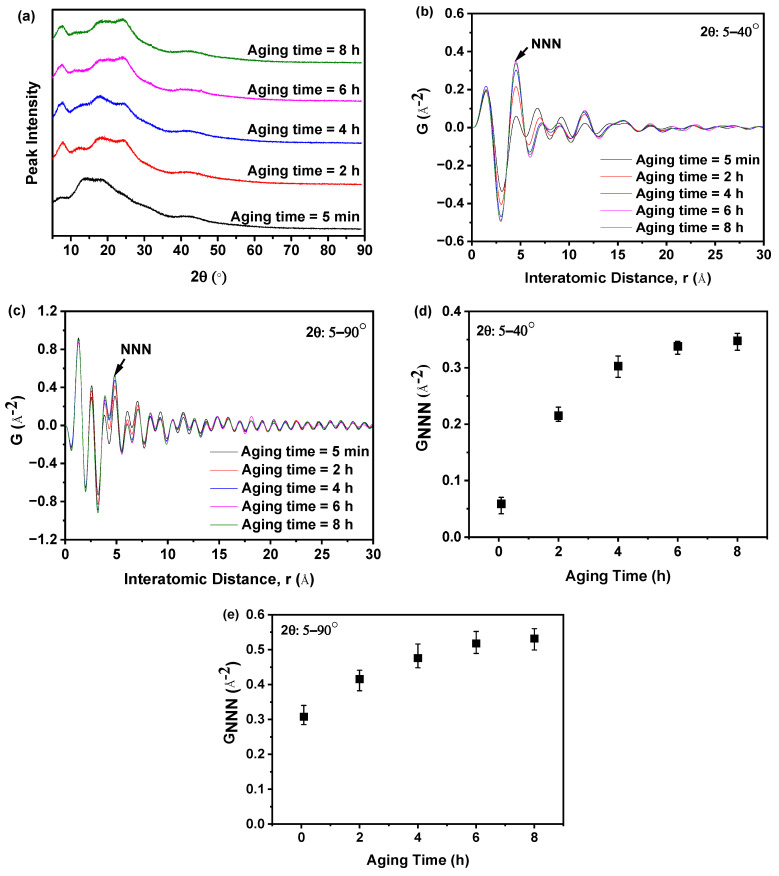
Effect of PXRD diffraction angle range on PDF analysis: (**a**) PXRD patterns of samples prepared under different aging times; (**b**) PDF (2θ = 5–40°); (**c**) PDF (2θ = 5–90°); (**d**) G_NNN_ (2θ = 5–40°, *n* = 3); and (**e**) G_NNN_ (2θ = 5–90°, *n* = 3).

To ascertain the optimal PXRD diffraction angle range for obtaining accurate information regarding the arrangement of nilotinib free base molecules through Fourier transformation of PXRD data into PDF data, this study conducted a comparison of PDF data obtained from different PXRD diffraction angle ranges. As demonstrated in Figure 6a, the PXRD patterns exhibited a single halo peak for all samples prepared under varying aging times. This observation suggested that the samples were in an amorphous state, and the distinctions between them could not be discerned solely based on their PXRD patterns. As shown in Figure 6b,c, it can be observed that as the aging time was increased from 5 min to 8 h, the G_NNN_ value exhibited a gradual increase. This suggested that a longer aging time has the potential to enhance the ordering of the nilotinib free base amorphous samples.

Additionally, by comparing Figure 6b,c, it is observed that the PDF data obtained from the Fourier transformation of the PXRD data measured with different PXRD diffraction angle ranges exhibit variations. When comparing the NNN peak intensities of the PDF, which was Fourier transformed from the range of 5–40°, with those of the range 5–90°, it become evident that the differences between amorphous samples were more pronounced. This phenomenon suggested that 5–90° exhibited a higher level of noise signals compared to 5–40°. Consequently, this can lead to the presence of inaccurate information during the analysis of the PDF data. This phenomenon has also been observed by other researchers, who have shown that the quality of the end-measurement data determined the effective PXRD diffraction angle range (from 2θmin to 2θmax) during the Fourier transformation from PXRD data to PDF data [35,36,37].

In addition, a lower G_NNN_ value indicated a more disordered arrangement of molecules in the solid structure. As shown in Figure 6d,e, when 5–90° was used, the G_NNN_ values varied less among samples (0.308–0.532); while when 5–40° was used, the G_NNN_ values varied more among samples (0.059–0.348). This phenomenon suggested that the Fourier transformation of PXRD data measured within the range of 5–40° into PDF was more sensitive to changes in the amorphous solid structure, as compared to the range of 5–90°. Therefore, a PXRD diffraction angle range of 5–40° is recommended to analyze the solid form of nilotinib free base by PDF accurately. These findings were consistent with the conclusion drawn in the previous studies conducted by Dykhne et al., where they suggested that a narrower PXRD diffraction angle range should be used to mitigate the adverse signal-to-noise ratio observed at high diffraction angles [38].

### 2.4. Calibration of Measurement Techniques

**Figure 7:**
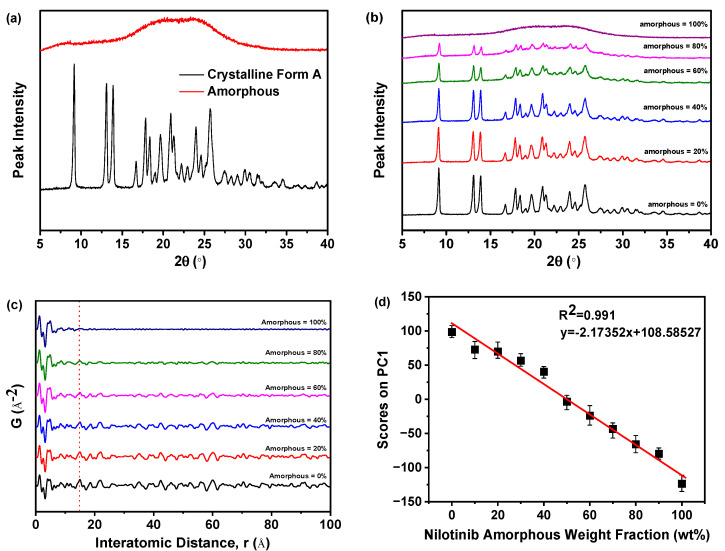
Calibration of measurement techniques: (**a**) PXRD patterns of the nilotinib free base pure crystalline Form A and pure amorphous form; (**b**) PXRD patterns of the mixture samples (PXRD diffraction angle range was 5–40°, PXRD detector was semiconductor detector); (**c**) PDF (r = 0–15 Å); (**d**) calibration of nilotinib free base amorphous weight fraction with PCA scores on PC1 (r = 0–15 Å, *n* = 3).

As presented in Figure 7a, the PXRD patterns of the pure crystalline Form A of nilotinib free base exhibited distinct peaks at 9.07°, 13.08°, 13.82°, 16.68°, 17.85°, 18.26°, 20.84°, 21.39°, 24.11°, and 25.22°. This observation was in complete accordance with the characteristic peaks exhibited by nilotinib crystalline Form A, as documented in prior research studies [39,40]. However, the amorphous form exhibited only a halo peak. As depicted in Figure 7b, the PXRD patterns demonstrated that the characteristic peak height of crystalline Form A gradually diminished and eventually disappeared entirely with the progressive increase in the weight fraction of the amorphous form of nilotinib free base in the mixed samples. This observation suggested a corresponding increase in intermolecular disorder.

As revealed in Figure 7c, the PDF of each sample exhibited no significant variation in the interatomic distance when it was below 14.35 Å. The PDF of each sample exhibited a notable disparity in the interatomic distance, specifically above 14.35 Å. As the proportion of amorphous form in the sample increased incrementally, the fluctuation in the PDF decreased. This finding indicated that PDFs with interatomic distances less than 14.35 Å encompassed both intramolecular and intermolecular structural details of the nilotinib free base. Consistent with the longest intramolecular interatomic distance (14.35 Å) of nilotinib free base, as shown in Figure 1b. However, the PDF data with a distance greater than 14.35 Å exclusively encompassed intermolecular structural information. At the same time, this also implied that the disorder in the sample gradually increased as the weight fraction of amorphous material increased.

In addition, the PDF interatomic distance range of 0–15 Å was utilized to calibrate the PCA analysis. The study revealed a negative correlation between the amorphous weight fraction of nilotinib free base and the PCA scores on PC1. As the amorphous weight fraction increased, the PCA scores on PC1 decreased. The correlation between the amorphous weight fraction in the mixed samples and the PCA scores on PC1 was found to be significant (R^2^ = 0.991), indicating a strong linear relationship (Figure 7d). This phenomenon illustrated that by employing optimized conditions for assessing the degree of disorder and physical stability in amorphous nilotinib free base, more reliable data can be obtained for PXRD, PDF, and PCA. These optimized conditions included a PXRD diffraction angle range of 5–40°, the use of a semiconductor PXRD detector, and a PDF interatomic distance range of 0–15 Å. Thus, a more precise and expedited evaluation of the degree of disorder and physical stability in an amorphous nilotinib free base can be achieved.

### 2.5. Accelerated Stability Test

**Figure 8:**
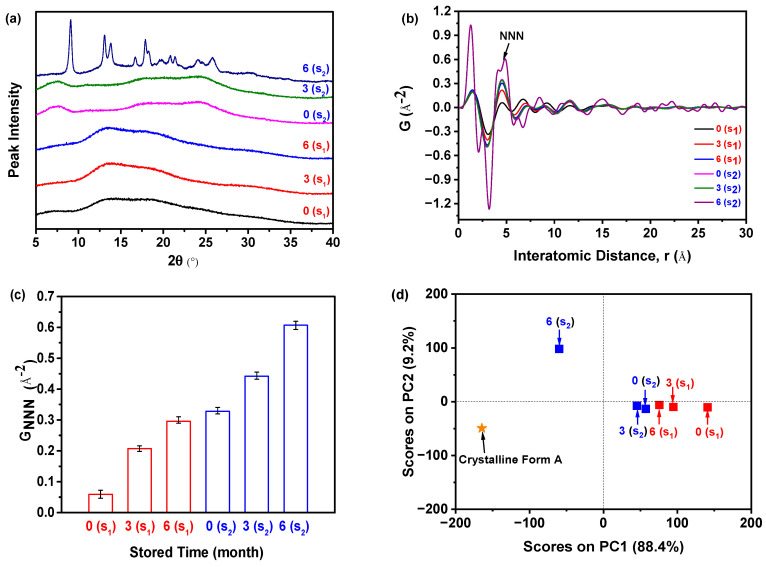
Accelerated stability test: (**a**) PXRD patterns of the samples S_1_ and S_2_ with different storage times in the drug stability test chamber (PXRD diffraction angle range was 5–40°, PXRD detector was semiconductor detector); (**b**) PDF; (**c**) G_NNN_ (*n* = 3); (**d**) PCA (r = 0–15 Å).

As illustrated in Figure 8a, the PXRD patterns demonstrated that sample S_1_, which was aged for 5 min, and stored for 0, 3, and 6 months, exhibited only a halo peak. This indicated that the sample remained in its amorphous form and did not undergo any crystallization during the accelerated stability test. On the contrary, in the case of sample S_2_ (aged for 8 h) stored for 0 and 3 months, the patterns similarly displayed solely a halo peak, suggesting that they maintained their amorphous state. However, after storing sample S_2_ for 6 months, the resulting pattern displayed the distinctive peak associated with the crystalline Form A of nilotinib free base. This peak was observed at angles of 9.07°, 13.08°, 13.82°, 16.68°, 17.85°, 18.26°, 20.84°, 21.39°, 24.11°, and 25.22°. The characteristic peaks observed in this study were found to be in complete agreement with the characteristic peaks previously reported for crystalline Form A of nilotinib [39,40]. It is important to highlight that, following a storage period of 6 months, the amorphous sample S_2_ had undergone partial transformation into crystalline Form A. However, a significant portion of the sample remained in its amorphous state. As a result, the PXRD pattern of this sample closely resembled that of the sample shown in Figure 7b. According to the measurement calibration in Figure 7d, it was calculated that the amorphous content was 78%, and the crystalline Form A content was 22% in this sample. This finding indicated that sample S_2_ experienced a phase transformation from its amorphous state to crystalline Form A as the storage time was increased from 3 to 6 months. Accordingly, it was revealed that sample S_1_ exhibited greater physical stability compared to sample S_2_.

However, discerning the distinctions among the amorphous samples solely based on their PXRD patterns proved to be a challenging task. Therefore, the PDF was utilized, as displayed in Figure 8b. The findings of the study revealed that sample S_2_, which was stored for 6 months, exhibited greater fluctuations in PDF and the highest peak height of NNN in comparison to the remaining samples. Additionally, the NNN peak height of the amorphous samples in S_1_ exhibited an increase as the storage time increased from 0 to 6 months. Similarly, the NNN peak height of the amorphous samples in S_2_ showed an increase as the storage time increased from 0 to 3 months.

In addition, the degree of disorder in the samples can also be assessed by utilizing the G_NNN_ value, as illustrated in Figure 8c. For samples S_1_ and S_2_, it was observed that the G_NNN_ value of the sample stored for 6 months was higher compared to those stored for 0 and 3 months. This suggested that a lower G_NNN_ value was indicative of a higher degree of disorder. The sample stored for 0 months exhibited the lowest G_NNN_ value, indicating a higher degree of disorder. Furthermore, upon comparing samples S_1_ and S_2_, both of which were stored for 0 months, it was observed that sample S_1_ exhibited a lower G_NNN_ value. This suggested that sample S_1_ possessed a higher degree of disorder and superior physical stability in comparison to sample S_2_. Hence, it can be observed that sample S_2_ exhibited a higher degree of ease in crystallization compared to sample S_1_ (Figure 8a).

The PCA analysis was conducted using the PDF data to evaluate the degree of disorder, as shown in Figure 8d. The samples were categorized into three groups based on the observations made: (1) the crystalline Form A, which served as the reference; (2) sample S_2,_ which had been stored for 6 months; and (3) the remaining samples. Sample S_2_, which had been stored for 6 months, was found to be positioned separately from the other samples along the PC1 axis and was close to the crystalline Form A. This suggested that it exhibited a higher degree of similarity to the crystalline Form A compared to the other samples. Among the samples belonging to the third category, it was observed that sample S_1_, which had been stored for 0 months, exhibited the greatest deviation from the crystalline Form A. This suggested that it possessed the most distinct solid structure compared to the crystalline Form A and exhibited the highest degree of disorder. Specifically, it can be observed that sample S_1_, which was stored for 0 months, exhibited greater physical stability compared to sample S_2_, which was also stored for 0 months. According to the results of the PCA analysis, it has been confirmed that sample S_1_ exhibited greater physical stability compared to sample S_2_.

In conclusion, the findings of this study suggest that there was no need to wait for 6 months to assess the physical stability in samples through PXRD measurements. Instead, the PXRD measurement of the samples during the initial stage of sample preparation, in conjunction with the analysis of the PDF and PCA, can effectively and expeditiously evaluate the degree of disorder and physical stability in amorphous drugs. Hence, the utilization of PXRD, PDF, and PCA techniques offer significant benefits in the precise and efficient evaluation of the degree of disorder and physical stability in amorphous drugs, as compared to an accelerated stability test. Moreover, by using this method, we have quickly and accurately evaluated the amorphous solids’ physical stability in nilotinib free base, which were prepared under different precipitation conditions [41,42,43].

## 3. Materials and Methods

### 3.1. Materials

**Figure 9:**
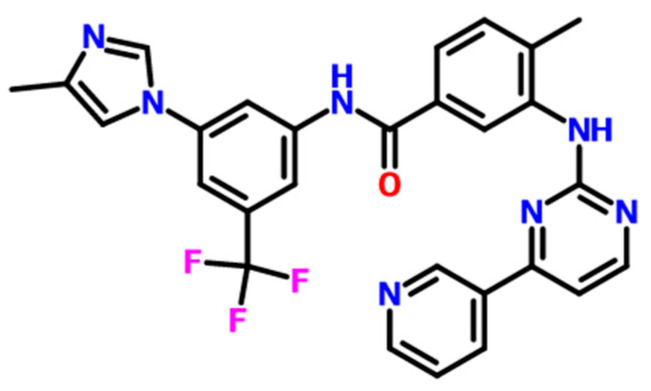
The molecular structure of nilotinib free base, C_28_H_22_F_3_N_7_O.

Heryi Pharma provided nilotinib free base (purity ≥ 98%, crystalline Form A, Anhui Heryi Pharmaceutical Co., Ltd., Tianchang, China), and the molecular structure of nilotinib free base is shown in Figure 9. HCl and NaOH were purchased from Titan (37% and purity ≥ 99%, Shanghai Titan Technology Co., Ltd., Shanghai, China). Deionized water was obtained with a Millipore ultrapure water system (Applied Membranes Inc., Vista, CA, USA).

### 3.2. Preparation of Nilotinib Free Base Amorphous Samples

**Figure 10:**
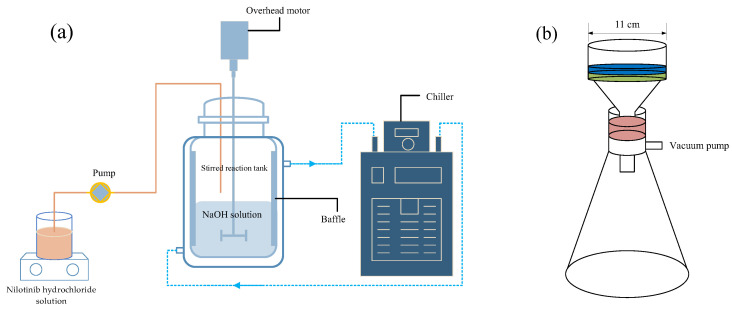
(**a**) Illustrated the experimental setup used to precipitate the amorphous form of nilotinib free base; (**b**) The process involved filtration of the suspension using a circulating water vacuum pump.

As depicted in Figure 10a, a suitable quantity of nilotinib free base solid was completely dissolved in 2.0 mol/L HCl to yield a 100 mL solution of nilotinib hydrochloride with a concentration of 0.126 mol/L. The solution mentioned above underwent filtration using qualitative filter paper (TY3-0005, Shanghai Titan Technology Co., Ltd., Shanghai, China) to eliminate any solid impurities. This process resulted in the acquisition of a transparent solution of nilotinib hydrochloride. Subsequently, a stirring reaction tank equipped with four baffles (S300, Beijing Century Senlang Experimental Instrument Co., Ltd., Beijing, China) was filled with 100 mL of NaOH solution (2.0 mol/L). The NaOH solution was cooled to a temperature of 5 °C using a chiller (KGDH-2030, Nanjing Kenfan Electronic Technology Co., Ltd., Nanjing, China). Under a stirring speed of 1000 rpm, the nilotinib hydrochloride solution, as previously described, was introduced into the NaOH solution in the stirring reaction tank using a peristaltic pump (BT100FC, Baoding Rongbai Constant Flow Pump Manufacturing Co., Ltd., Baoding, China) at a consistent feed rate of 22 mL/min.

After the mixing and subsequent neutralization of the two solutions, a solid suspension comprising amorphous particles of nilotinib free base was promptly precipitated. The suspension underwent continuous stirring for specific durations (5 min, 2 h, 4 h, 6 h, and 8 h, also called aging time) following the completion of feeding. A circulating water vacuum pump (SHZ-III A, Gongyi Ruide Instrument Equipment Co., Ltd., Gongyi, China) was employed in combination with qualitative filter paper to enhance the efficiency of the filtration process for the suspension’s filtrate, leading to the collection of the wet cake of the nilotinib free base (Figure 10b). During the filtration process, the wet cake underwent a thorough washing using 100 mL of deionized water. The washed wet cake was subsequently subjected to drying in a vacuum oven (DHG-9055A, Wujiang Yonglian Machinery Equipment Factory, Wujiang, China) at a temperature of 40 °C for 18 h. This process resulted in the production of nilotinib free base amorphous dry powder samples, which exhibited a water content of approximately 3.5%. The samples were subsequently transferred into glass bottles with caps and stored at ambient temperature before measurement.

### 3.3. Powder X-ray Diffraction

The nilotinib free base samples were promptly measured at room temperature using PXRD with two distinct types of detectors. These detectors included the semiconductor detectors equipped in the Bruker D8 and Bruker D2 powder X-ray diffractometer (Bruker in Karlsruhe, Germany), as well as the scintillation detectors equipped in the Rigaku Miniflex600 and Rigaku Smartlab powder X-ray diffractometer (Rigaku in Tokyo, Japan). The PXRD measurement parameters utilized in this study were as follows: Ag Kα radiation with a wavelength of 0.56 Å, a tube voltage of 100 kV, and a tube current of 80 mA. The scanning step size was maintained at a constant value of 0.02°, while the scanning speed was set at 6°/min, and time per step was set at 1 s. Moreover, the divergent slit was set at 0.6 mm, the anti-scatter slit was set at 3 mm. The specific PXRD diffraction angle range for the scans was set at 5–40° or 5–90°. The instrument continuously performed scans on the samples of nilotinib free base, allowing for the acquisition of PXRD patterns for each sample. In addition, it should be noted that two Rigaku Smartlab powder X-ray diffractometers were utilized in this study. These diffractometers were sourced from different universities and have been designated as Smartlab-1 and Smartlab-2 for the purposes of this research. Each sample underwent three measurements.

### 3.4. Pair Distribution Function

The software PDFgetX2 (version 1.0), which is freely available, was utilized for Fourier transforming PXRD data to obtain the PDF data. The program was developed by Qiu et al., and the details of its setup were previously described [44]. The program can be accessed via the following URL: https://web.pa.msu.edu/cmp/billinge-group/programs/PDFgetX2/ (accessed on 6 March 2024).

### 3.5. Principal Components Analysis

This study employed PCA to analyze and interpret the differences observed in the PXRD patterns and their corresponding PDF data. As outlined in the study conducted by Karmwar et al., the preprocessing and scaling of the data were carried out using SIMCA 15 (Ver. 15.0, Sartorius, Göttingen, Germany) [19]. During the PCA analysis, a specific PDF interatomic distance range was selected. The ranges chosen for PCA analysis were 0–15 Å or 15–100 Å. Subsequently, the PCA data were obtained for each amorphous sample.

### 3.6. Calibration Experiment

Eleven mixtures were prepared, these samples were physically mixed based on the weight fractions in their respective phases, consisting of a combination of nilotinib free base amorphous form solid and crystalline Form A solid. The weight fraction of the amorphous form solid in these mixtures ranged from 0% to 100%. A PXRD measurement was conducted on each mixture using a Bruker D8 powder X-ray diffractometer equipped with a semiconductor detector. A PXRD diffraction angle range of 5–40° was employed for the measurement. The PXRD data obtained from the experiment were subsequently subjected to Fourier transformation to generate the PDF data. Finally, a PDF interatomic distance range of 0–15 Å was utilized to conduct the PCA analysis. The PCA data were utilized for the calibration of the amorphous solid mass percentage in the mixture. Each experiment was conducted in triplicate.

### 3.7. Accelerated Stability Test

Two distinct amorphous samples of nilotinib free base, aged for 5 min and 8 h, were chosen for the accelerated stability test. The sample aged for 5 min was labelled as S_1_, while the sample aged for 8 h was labelled as S_2_. Additionally, following the drying process of the amorphous samples of nilotinib free base, each sample was divided into three equal parts. One part of the samples was promptly subjected to a PXRD measurement (referred to as 0 (S_1_) and 0 (S_2_), representing a storage duration of 0 months). Furthermore, the remaining four portions of the samples were promptly transferred into glass bottles without caps and subsequently stored in a drug stability test chamber (YP-250GSP, Shanghai Suying Test Instrument Co., LTD, Shanghai, China) for a duration of 3 and 6 months. These samples were designated as 3 (S_1_), 6 (S_1_), 3 (S_2_), and 6 (S_2_), respectively. The temperature and relative humidity within the chamber were maintained at 40 ℃ and 75%, respectively. Following the prescribed procedure outlined in Section 3.6, the PXRD, PDF, and PCA data of these samples were acquired after the storage periods. Each experiment was conducted in triplicate.

### 3.8. Thermogravimetric Analysis

The water content in the dried samples was determined using TGA conducted on a TGA8000 analyzer (PerkinElmer, Waltham, MA, USA). Approximately 5 mg of each sample was placed in an aluminum pan with an open lid. The temperature was increased from 30 to 180 °C at a heating rate of 10 °C/min, while nitrogen was purged at a flow rate of 20 mL/min. To ensure accuracy, each test was repeated three times.

### 3.9. Summary of All Used Samples

In summary, as shown in Table 1, our study prepared five amorphous solid samples aged for durations of 5 min, 2 h, 4 h, 6 h, and 8 h, labeled sequentially from 1 to 5. Additionally, for the accelerated stability test, samples aged for 5 min and 8 h were selected for comparison. Each sample was then segmented into three parts and stored for 0 months, 3 months, and 6 months before being subjected to PXRD measurement, as detailed in Table 1 (samples numbered 6–11).

## 4. Conclusions

The assessment of a drug’s physical stability plays a pivotal role in determining its duration of effectiveness during storage. A novel methodology was employed in this research to expedite and ensure precise evaluation of the physical stability in amorphous drugs. The present study developed a system utilizing PXRD, PDF, and PCA to quantify the degree of disorder and evaluate the physical stability in amorphous drugs. The initial description of the molecular structure of crystalline Form A in the antitumor drug nilotinib free base was provided. Subsequently, the amorphous samples of nilotinib free base were subjected to a PXRD measurement to determine their properties. The degree of disorder and physical stability in the nilotinib free base amorphous samples were then evaluated through a combination of PDF and PCA analyses. This novel approach demonstrated enhanced speed and precision compared to traditional techniques used for evaluating the physical stability in drugs.

The study additionally identified the key factors that influence the precision of the assessment methodology. The factors considered in this study were the PDF interatomic distance range, the type of detector used in the PXRD, and the PXRD diffraction angle range. The determination of optimal conditions for evaluating the degree of disorder and physical stability in amorphous drugs involved several factors. Firstly, it was found that a PDF interatomic distance range of 0–15 Å provided more reliable data for PCA analysis. Secondly, the utilization of a semiconductor detector in PXRD measurement facilitated the acquisition of more precise PDF data. Lastly, a PXRD diffraction angle range of 5–40° was found to be crucial in accurately predicting the molecular arrangement information of the amorphous drugs.

In conclusion, the system outlined in this study served as a crucial resource for investigating the degree of disorder and physical stability exhibited by amorphous drugs. However, it is important to acknowledge that relying solely on a physical stability assessment is insufficient for evaluating the formulation stability; chemical stability must also be taken into account. The objective of this study was to develop and optimize a technology for assessing the physical stability in amorphous drugs. So, future research will focus on investigating the chemical and physical stability in nilotinib free base and other drugs, while simultaneously optimizing the conditions for preparing amorphous formulations.

## Figures and Tables

**Table 1:** Summary of all used samples in this study.

Numbers	Samples Name	Preparation and Measurement Conditions
1	5 min	Aged for 5 min and then immediately measured
2	2 h	Aged for 2 h and then immediately measured
3	4 h	Aged for 4 h and then immediately measured
4	6 h	Aged for 6 h and then immediately measured
5	8 h	Aged for 8 h and then immediately measured
6	0 (S_1_)	Aged for 5 min and then immediately measured
7	3 (S_1_)	Aged for 5 min and then measured after stored for 3 months
8	6 (S_1_)	Aged for 5 min and then measured after stored for 6 months
9	0 (S_2_)	Aged for 8 h and then immediately measured
10	3 (S_2_)	Aged for 8 h and then measured after stored for 3 months
11	6 (S_2_)	Aged for 8 h and then measured after stored for 6 months

## Data Availability

Data are contained within the article.
